# Tissue-Resolved Metabolomic Profiling of Ten *Prunus salicina* × *P. armeniaca* Cultivars Reveals Peel–Flesh Metabolic Differences Associated with Fruit Color and Antioxidant Capacity

**DOI:** 10.3390/foods15162790

**Published:** 2026-08-09

**Authors:** Taishan Li, Menghan Wu, Yanke Geng, Gaigai Du

**Affiliations:** 1 Research Institute of Non-Timber Forestry, Chinese Academy of Forestry, Zhengzhou 450003, China; 2 Research Institute of Forestry, Chinese Academy of Forestry, Beijing 100091, China

**Keywords:** interspecific hybrid, widely targeted UPLC-MS/MS, machine learning, flavonoid glycosides, anthocyanin accumulation, fruit quality evaluation

## Abstract

*Prunus salicina* × *P. armeniaca*, an interspecific hybrid of Chinese plum and apricot, shows substantial variation in peel and flesh color and quality traits. Ten cultivars were characterized by integrating physicochemical measurements, spectrophotometric assays of phytochemical content and antioxidant capacity, and widely targeted ultra-performance liquid chromatography-tandem mass spectrometry (UPLC-MS/MS) metabolite profiling. Tissue type was the main source of chemical variation. Peel contained higher total phenolic (TPC), flavonoid (TFC), and anthocyanin (TAC) contents and greater 2,2-diphenyl-1-picrylhydrazyl (DPPH) radical-scavenging capacity, 2,2′-azino-bis (3-ethylbenzothiazoline-6-sulfonic acid) (ABTS) radical-scavenging capacity, and ferric reducing antioxidant power (FRAP), whereas flesh showed higher relative abundances of sugars, sugar alcohols, organic acids, amino acid derivatives, lipids, nucleotides, and terpenoids. Among 248 annotated metabolites, flavonoids were predominant, and color-related differentially accumulated metabolites were mainly enriched in phenylpropanoid and flavonoid pathways, particularly in purple–yellow comparisons. Integrating tissue-specific correlations, random forest models, and color-gradient patterns prioritized cyanidin-3-O-glucoside, dihydromyricetin, genistin, and orientin as cross-tissue color-associated metabolites. Cyanidin-3-O-glucoside showed the strongest association with coloration and anthocyanin content, whereas 4′-O-glucosylvitexin was strongly connected with phenolic, flavonoid, anthocyanin, and antioxidant traits. Comprehensive nutritional quality index rankings further distinguished cultivar-specific peel and flesh quality profiles. These results provide a tissue-resolved metabolic basis for evaluating and utilizing plum–apricot hybrid germplasm.

## 1. Introduction

Among stone fruits, *Prunus salicina* × *P. armeniaca* is a useful hybrid system because plum-like and apricot-like traits occur in the same fruit background. The cultivars differ in peel and flesh color, texture, aroma, and sugar–acid balance, giving a compact panel for examining eating quality and functional value. Market quality is shaped by size, soluble solids, organic acids, firmness, color, and aroma [1]. In addition to these basic quality traits, metabolites such as phenolic acids, anthocyanins, other flavonoids, and terpenoids collectively contribute to fruit coloration, antioxidant potential, and aroma [2].

Peel and flesh need to be considered separately in this hybrid. Because the peel is directly exposed to light, temperature fluctuations, mechanical injury, and pathogen pressure, these environmental and biotic factors can stimulate the accumulation of protective phenolics, including anthocyanins, flavonols, phenolic acids, and proanthocyanidins [3,4]. Flesh, by contrast, determines much of the edible portion and contains compounds tied to juiciness, texture, sweetness, acidity, and flavor, including sugars, organic acids, amino acids, and water-soluble constituents [5]. A tissue-resolved analysis is therefore required before quality variation in *Prunus salicina* × *P. armeniaca* can be interpreted.

Color provides a practical entry point for separating metabolic types in plum–apricot hybrids. Red and purple tissues usually contain abundant anthocyanins and flavonoids, whereas yellow tissues may reflect lower anthocyanin levels or different carotenoid, chlorophyll, and flavonoid profiles [6,7]. In peach, apple, and plum, red coloration has been associated with anthocyanins, flavonoids, and phenolic acids [8,9,10,11]. Antioxidant capacity may follow these color-linked changes, but it is not governed by anthocyanins alone. Flavonols, flavanols, phenolic acids, and other phenolics also contribute to radical-scavenging and reducing activity [12].

It remains unclear how peel and flesh color classes in *Prunus salicina* × *P. armeniaca* differ when basic quality, phytochemical content, antioxidant capacity, and UPLC-MS/MS metabolite profiles are evaluated together. Most stone-fruit evidence still comes from plum, apricot, peach, or apple, and tissue-resolved data for this hybrid are scarce. We therefore analyzed ten cultivars with contrasting peel and flesh colors. The analysis compared quality and antioxidant traits among tissue-color groups, profiled metabolites separately in peel and flesh, screened color- and antioxidant-associated metabolites, and used a comprehensive nutritional quality index to rank cultivars. This study links color-resolved food chemistry with germplasm evaluation.

## 2. Materials and Methods

### 2.1. Plant Materials and Sample Collection

Ten plum–apricot hybrid (*Prunus salicina* × *P. armeniaca*) cultivars were sampled: ‘FWHH’ (‘FengWeiHuangHou’), ‘WJ’ (‘WeiJin’), ‘WW’ (‘WeiWang’), ‘KLD’ (‘KongLongDan’), ‘WD’ (‘WeiDi’), ‘XL02’ (‘XingLi02’), ‘XL01’ (‘XingLi01’), ‘WX’ (‘WeiXin’), ‘WH’ (‘WeiHou’), and ‘FWMG’ (‘FengWeiMeiGui’). All cultivars were grown at the Yuanyang Experimental Base of the Research Institute of Non-timber Forestry, Chinese Academy of Forestry, Yuanyang County, Henan Province, China (34°55′–34°56′ N, 113°46′–113°48′ E). The trees were subjected to consistent irrigation, fertilization, pruning, and pest-management practices. During the growing season from March to August 2025, the mean air temperature was 23.9 °C. The mean daily minimum and maximum temperatures were 18.4 °C and 29.6 °C, respectively. Cumulative precipitation and sunshine duration during this period were 491.1 mm and 1949.5 h, respectively. Meteorological estimates for the experimental-site coordinates were obtained from the Open-Meteo Historical Weather API using ERA5/ERA5-Land reanalysis data (accessed 20 July 2026). Fruits of all cultivars were harvested at commercial maturity in 2025, with the cultivar-specific harvest dates provided in Table S1. For each cultivar, three trees with similar growth status were chosen as biological replicates. From each tree, ten similarly sized fruits were collected at random; fruits showing mechanical injury, pest damage, or disease symptoms were discarded. Peel and flesh were separated immediately, frozen in liquid nitrogen, and kept at −80 °C before analysis. The ten fruits collected from each tree were pooled to form one biological replicate, resulting in three biological replicates per cultivar.

### 2.2. Fruit Morphology, Basic Quality Traits, and Color Measurements

Fruit weight was measured with an electronic balance. Longitudinal and transverse diameters were measured with a digital caliper, and fruit shape index was calculated as transverse diameter/longitudinal diameter. Total soluble solids (TSS) were read with a portable refractometer and expressed as °Brix. Titratable acidity (TA) followed the acid-base titration procedure according to Baccichet et al. [13] and was expressed as malic acid equivalents (g/100 g FW). The TSS/TA ratio was then used to describe sugar–acid balance. Juice pH was measured with a pH meter. Firmness was determined according to Qiu et al. [14] with a texture analyzer fitted with a 2 mm cylindrical probe; the probe punctured the equatorial region, and the maximum force was recorded in newtons (N). Peel and flesh color were measured using an NH310 colorimeter (ThreeNH Technology Co., Ltd., Shenzhen, China) in the CIELAB system. Five readings per sample were averaged for L*, a*, and b*. Chroma was calculated as C* = (a*^2^ + b*^2^)^1/2^, and hue angle was calculated as h° = atan2(b*, a*).

### 2.3. Phenolic, Flavonoid, Anthocyanin, and Antioxidant Assays

For phytochemical extraction, peel and flesh were ground under liquid nitrogen, and 0.5 g of powder was combined with 5 mL of 70% methanol. The mixture was vortexed, sonicated for 30 min, and centrifuged at 8000 rpm for 15 min at 4 °C. The supernatant served as the shared extract for the phytochemical and antioxidant assays. No internal standard was added to this shared extract because TPC, TFC, and TAC were determined by spectrophotometric equivalence methods.

Total phenolic content (TPC), total flavonoid content (TFC), and total anthocyanin content (TAC) were determined using the Folin–Ciocalteu, aluminum chloride, and pH differential methods, respectively. For TPC, 200 μL of extract was mixed with 1 mL of Folin–Ciocalteu reagent and 0.8 mL of 7.5% Na_2_CO_3_, incubated in the dark at room temperature for 30 min, and measured at 765 nm [15]. For TFC, 200 μL of extract was mixed with 1 mL of distilled water and 75 μL of 5% NaNO_2_. After 5 min, 150 μL of 10% AlCl_3_ was added, followed 6 min later by 500 μL of 1 M NaOH, and absorbance was measured at 510 nm [16]. For TAC, 1 mL of extract was separately diluted to 10 mL with pH 1.0 buffer (0.025 M KCl) and pH 4.5 buffer (0.4 M sodium acetate), equilibrated for 20 min, and measured at 520 and 700 nm [17]. TPC, TFC, and TAC were expressed as mg gallic acid equivalents (GAE), catechin equivalents (CE), and cyanidin-3-O-glucoside equivalents (C3GE) per 100 g fresh weight, respectively.

DPPH, ABTS, and FRAP capacities were determined according to established methods [18,19,20]. For DPPH, 100 μL of extract was mixed with 3.9 mL of 0.1 mM DPPH in methanol, incubated in the dark for 30 min, and measured at 517 nm [18]. The ABTS radical cation solution was generated by reacting 7 mM ABTS with 2.45 mM K_2_S_2_O_8_ in the dark at room temperature for 12–16 h and was subsequently diluted to an absorbance of 0.70 ± 0.02 at 734 nm. Extract (50 μL) was mixed with 4 mL of the ABTS working solution, reacted for 6 min, and measured at 734 nm [19]. For FRAP, the working reagent was prepared by mixing 300 mM acetate buffer (pH 3.6), 10 mM TPTZ, and 20 mM FeCl_3_ at a ratio of 10:1:1. Extract (100 μL) was mixed with 3 mL of the FRAP reagent, incubated at 37 °C for 10 min, and measured at 593 nm [20]. Antioxidant capacities were calculated using Trolox calibration curves and expressed as μmol Trolox equivalents per gram fresh weight (μmol TE/g FW). All assays included three biological replicates, each measured in four technical replicates.

### 2.4. Metabolite Extraction and UPLC-MS/MS Profiling

For UPLC-MS/MS profiling, frozen peel and flesh were ground in liquid nitrogen. A 50 mg aliquot of each powder sample was extracted in 1.0 mL of chilled 70% aqueous methanol containing 0.1 mg/L lidocaine, which served as the internal standard for extraction and instrument stability. The extract was vortexed and centrifuged at 12,000× *g* for 3 min at 4 °C. The supernatant was passed through a 0.22 μm membrane (ANPEL Laboratory Technologies (Shanghai) Inc., Shanghai, China) and transferred to vials for analysis.

An ACQUITY UPLC HSS T3 column (100 mm × 2.1 mm, 1.8 μm; Waters Corporation, Milford, MA, USA) separated the metabolites at 40 °C. The flow rate was 0.35 mL/min, and the injection volume was 2 μL. Mobile phase A consisted of water containing 0.1% formic acid, and mobile phase B consisted of acetonitrile containing 0.1% formic acid. The gradient elution program was as follows: 0–2.0 min, 5% B; 2.0–22.0 min, a linear increase from 5% to 95% B; 22.0–27.0 min, 95% B; 27.0–27.1 min, a decrease from 95% to 5% B; and 27.1–30.0 min, 5% B for column re-equilibration. An AB Sciex QTRAP 6500+ tandem mass spectrometer with an electrospray ionization source (SCIEX, Framingham, MA, USA) detected the metabolites. The instrument collected positive- and negative-ion data by multiple reaction monitoring (MRM). The transitions were based on the MWDB in-house database of MetWare Biotechnology Co., Ltd. (Wuhan, China). Analyst 1.6.3 software was used for data acquisition and processing. Metabolite annotation used retention time, precursor-to-product ion pairs, and diagnostic fragments. These data were compared with the MWDB and local databases. Peak areas were integrated for all detected metabolites, and metabolite abundance was expressed as relative peak area [21]. The dataset was used for relative rather than absolute quantification. Authentic standards and compound-specific calibration curves were not used for absolute quantification.

### 2.5. Differentially Accumulated Metabolites and Pathway Enrichment Analysis

Metabolite relative peak areas were log_2_(x + 1) before statistical analysis, where x represents the relative peak area. Tissue differences between peel and flesh were analyzed using a linear model, with cultivar included as a covariate to account for cultivar background effects. The limma package (version 3.52.0) fitted the differential models with empirical Bayes moderation [22]. The Benjamini–Hochberg (BH) method adjusted the *p* values for multiple tests. A metabolite was classified as differentially accumulated when its BH-adjusted *p* value was below 0.05 and its |log_2_FC| ≥ 1.

To investigate color-related metabolic differences, pairwise comparisons among purple, red, and yellow groups were performed separately in peel and flesh, including Purple vs. Red, Purple vs. Yellow, and Red vs. Yellow. The same limma-based differential analysis framework and significance thresholds were used for these color-group comparisons. For heatmap feature selection, an overall moderated F-test across three color groups was performed within each tissue. Metabolites were ranked according to the BH-adjusted *p* values from the overall test, and the top 40 metabolites were visualized. KEGG pathway enrichment and chemical category enrichment analyses of DAMs were performed using hypergeometric over-representation tests against the detected metabolite background, with BH-adjusted *p* < 0.05 considered significant.

### 2.6. Metabolite–Trait Correlation Analysis

Spearman rank tests examined the links between metabolite abundance and the CIELAB traits L*, a*, b*, C*, and *h*°. The same tests also included TPC, TFC, TAC, DPPH, ABTS, and FRAP. Correlation analysis was performed in R, and *p* values for all metabolite-trait combinations within each tissue were adjusted using the BH procedure. A correlation was considered significant when |ρ| was at least 0.70 and the BH-adjusted *p* value was below 0.05. In addition, a phenotype-anchored metabolite-trait correlation network was constructed using all peel and flesh samples and visualized using the igraph package (version 1.3.5) and Cytoscape software (version 3.9.1) [23].

### 2.7. Random Forest Model Construction and Predictive Feature Screening

Random forest models were constructed based on the metabolite relative abundance matrix to identify candidate metabolites associated with tissue type, color group, and FRAP values [24]. All biological replicates from the same cultivar were assigned to the same cross-validation fold to prevent information leakage between the training and test sets. Model performance was evaluated using repeated group-based 5-fold cross-validation with 20 repeats. Classification models were fitted using method = “rf”; the binary tissue-classification model was optimized using ROC and AUC was calculated from cross-validated prediction probabilities, whereas peel- and flesh-color models were trained separately and summarized using confusion matrices normalized by the true class. FRAP regression was performed using the ranger algorithm with permutation-based variable importance, and R^2^ and RMSE were calculated from cross-validated predictions. Candidate metabolites were prioritized according to their task-specific scaled importance scores and were interpreted as predictive features.

### 2.8. Construction of the Comprehensive Nutritional Quality Index

For CNQI calculation, peel and flesh were handled as separate evaluation units. Three components were used: edible quality index (EQI), phytochemical index (PI), and antioxidant potential index (API). EQI included cultivar-level TSS, TSS/TA, pH, firmness, and TA, with TA treated as a negative trait. PI used TPC, TFC, and TAC, whereas API used DPPH, ABTS, and FRAP. Each trait was first summarized at the Variety × Tissue level and then standardized within tissue by z-score transformation. The three subindices were weighted equally, and the final CNQI was again standardized within tissue to obtain CNQI_z for ranking.

### 2.9. Statistical Analysis and Data Visualization

Each measurement included three biological replicates. Kruskal–Wallis tests first assessed overall differences among color groups. Dunn’s post hoc tests then compared pairs of groups, and the BH method adjusted these *p* values. Planned Welch tests were used as sensitivity analyses for predefined pairwise color-group comparisons, and the corresponding BH-adjusted *p* values were retained in the source tables. Paired comparisons between peel and flesh from the same cultivar or biological replicate were performed using paired Wilcoxon signed-rank tests. For the selected metabolites, the data were log-transformed before one-way analysis of variance. Tukey’s honestly significant difference test then compared the color groups. R software (v 4.2.0) was used for all statistical analyses. The significance level was α = 0.05. Principal component analysis was performed using the prcomp function, and volcano plots, heatmaps, and other visualizations were generated using ggplot2 (version 3.4.2), pheatmap (version 1.0.12), and related R packages (version 4.2.2).

## 3. Results

### 3.1. Differences in Fruit Quality and Color Traits Among Cultivars

**Figure 1:**
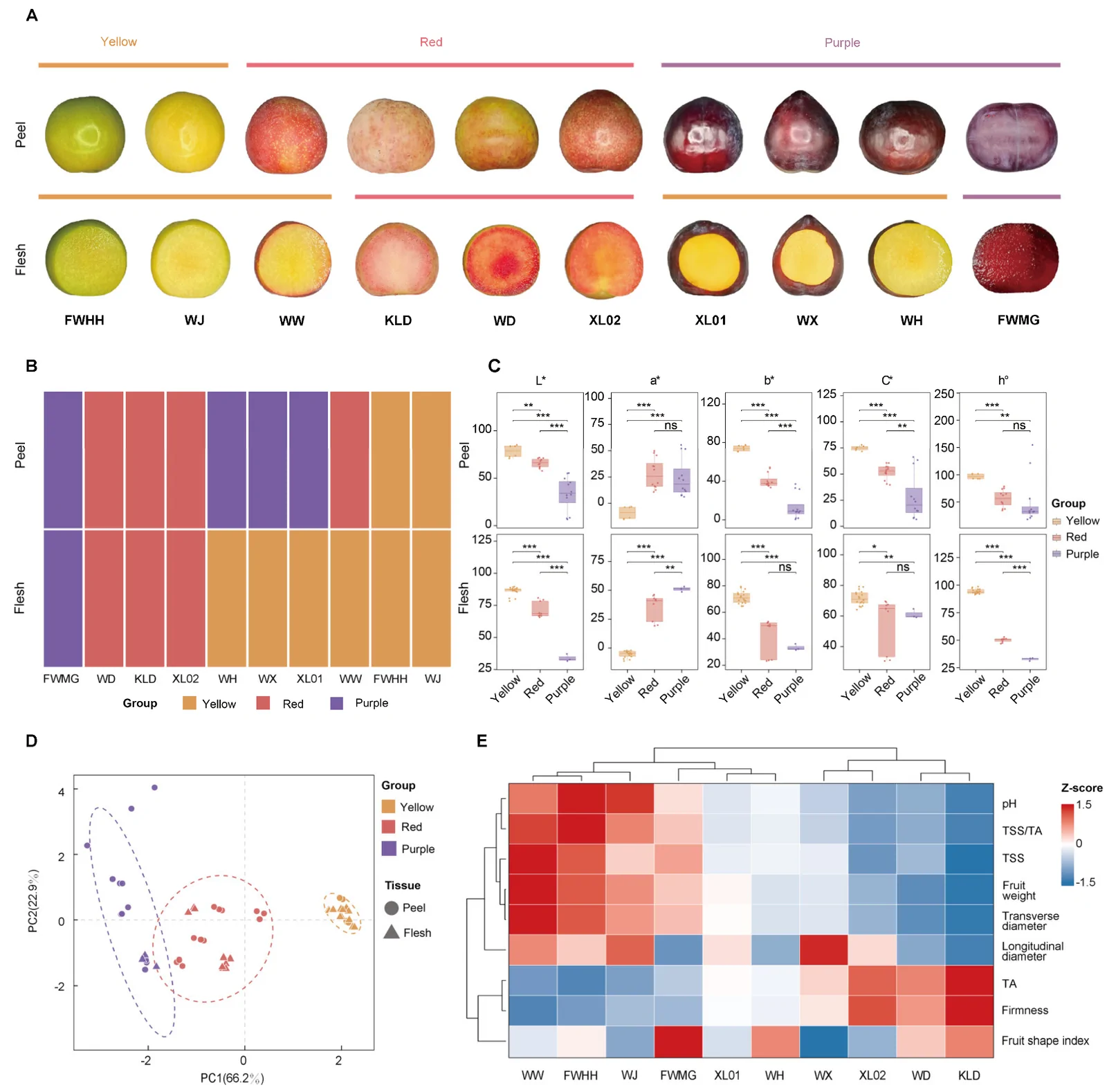
Fruit color classification and basic quality traits of 10 *Prunus salicina* × *P. armeniaca* cultivars. (**A**) Representative peel and flesh phenotypes of the 10 cultivars grouped by visual color class. (**B**) Color classification of peel and flesh samples. (**C**) CIELAB color parameters of peel and flesh among different groups. (**D**) Principal component analysis (PCA) based on CIELAB color parameters. Dashed ellipses indicate the 95% confidence regions for the three color groups. (**E**) Heatmap of fruit morphological and quality traits among cultivars. L*, lightness; a*, red-green coordinate; b*, yellow–blue coordinate; C*, chroma; *h*°, hue angle; TSS, total soluble solids; TA, titratable acidity. Asterisks indicate significant differences (*, *p* < 0.05; **, *p* < 0.01; ***, *p* < 0.001); ns, not significant. In (**E**), values were normalized by Z-score transformation.

In the ten *Prunus salicina* × *P. armeniaca* cultivars, peel and flesh color varied widely and were not always matched within the same fruit (Figure 1A,B). Visual scoring placed both tissue types into purple, red, or yellow groups. CIELAB measurements agreed with these groups (Figure 1C). Purple tissues had higher a* values and lower L*, b*, and *h*° values, yellow tissues had higher L*, b*, and *h*° values, and red tissues fell between these two color ranges. Principal component analysis of the CIELAB traits explained 89.1% of the variance with PC1 and PC2 and separated most samples by color type (Figure 1D). For later analyses, peel and flesh color classes were therefore treated independently.

Cultivars also differed in morphology and basic physicochemical traits (Figure 1E and Figure S1). Fruit weight was greater in ‘WW’, ‘FWHH’, and ‘WJ’ and lower in ‘KLD’ and ‘WD’. TSS was relatively high in ‘WW’, ‘FWHH’, and ‘FWMG’. The highest TA occurred in ‘KLD’, whereas ‘FWHH’ had the lowest TA, which gave higher TSS/TA ratios for ‘FWHH’ and ‘WW’ and lower ratios for ‘KLD’ and ‘XL02’. Firmness was greater in ‘KLD’ and ‘XL02’ and lower in ‘WW’ and ‘FWHH’. Overall, ‘WJ’, ‘FWHH’, and ‘WW’ combined larger fruit size, higher sugar–acid ratio, and softer texture, whereas ‘KLD’, ‘WD’, and ‘XL02’ were more acidic and firmer.

### 3.2. Differences in Bioactive Compounds and Antioxidant Capacity

**Figure 2:**
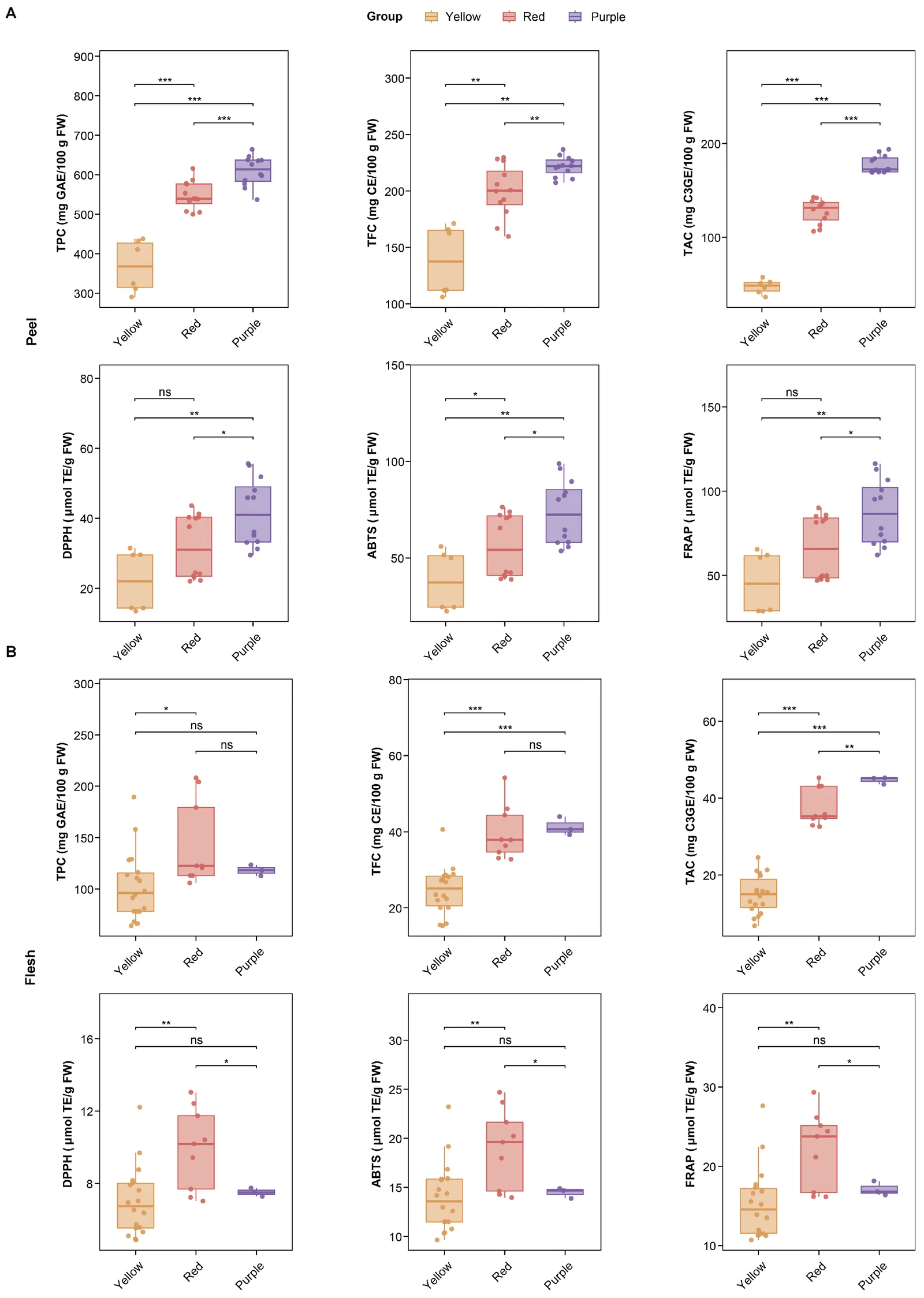
Phytochemical contents and antioxidant activities in peel and flesh of *Prunus salicina* × *P. armeniaca* fruits with different color classes. (**A**) Total phenolic content (TPC), total flavonoid content (TFC), total anthocyanin content (TAC), DPPH radical-scavenging activity, ABTS radical-scavenging activity and ferric reducing antioxidant power (FRAP) in peel. (**B**) Corresponding phytochemical contents and antioxidant activities in flesh. Asterisks indicate significant differences (*, *p* < 0.05; **, *p* < 0.01; ***, *p* < 0.001); ns, not significant. GAE, gallic acid equivalents; CE, catechin equivalents; C3GE, cyanidin-3-O-glucoside equivalents; TE, Trolox equivalents.

Tissue type and color class affected TPC, TFC, TAC, and the DPPH, ABTS, and FRAP results (Figure 2 and Figure S1). The peel had significantly higher values than the flesh for all six measures. In the peel, the accumulation of bioactive compounds and antioxidant capacity generally increased with deeper coloration, with ‘FWMG’ showing the highest values for all indicators.

Color-related differences were also observed in the flesh, but the patterns differed from those in the peel. ‘FWMG’ was the only cultivar in this study with both purple peel and purple flesh. ‘FWMG’ had slightly more TAC than the red-fleshed cultivars. However, its TPC and TFC were similar to those of the red-fleshed group. The red-fleshed group gave the highest DPPH, ABTS, and FRAP values. ‘FWMG’ and the yellow-fleshed group did not differ significantly. These results indicate that antioxidant capacity in the flesh is not determined solely by anthocyanin content, but may also be influenced by other phenolic, flavonoid, or non-phenolic antioxidant compounds.

Spearman correlation analysis further showed strong correlations among TPC/TFC/TAC and DPPH, ABTS, and FRAP in the peel, whereas the direct correlation between a* and antioxidant capacity was relatively weak (Figure S2). In flesh, higher a* values were linked to higher TAC, TFC, and antioxidant activity (Figure S3). In both peel and flesh, the correlations between TAC and antioxidant capacity were weaker than those between TPC/TFC and antioxidant capacity, suggesting that antioxidant potential is more likely determined by the overall composition of phenolic and flavonoid compounds rather than by the accumulation of anthocyanins alone.

### 3.3. Global Metabolomic Features of Peel and Flesh

**Figure 3:**
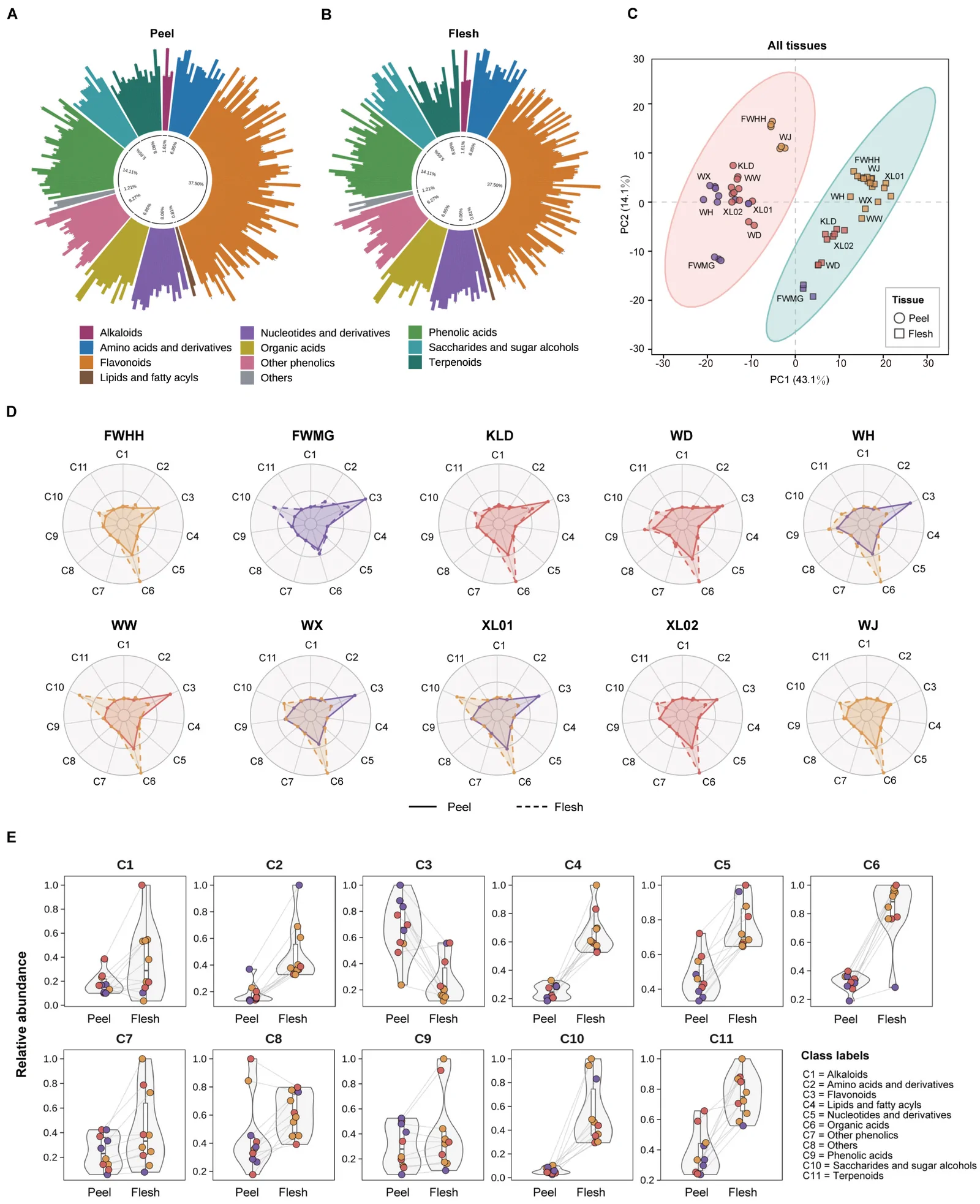
Global metabolomic profiles and tissue-specific distribution of metabolite classes in *Prunus salicina* × *P. armeniaca* peel and flesh. (**A**,**B**) Circular bar plots of the same 248 annotated metabolites in peel (**A**) and flesh (**B**). Metabolites are arranged in the same order and grouped by chemical class in both panels; therefore, sector widths and class percentages are identical, whereas radial bar lengths represent tissue-specific relative abundance. (**C**) Principal component analysis (PCA) of UPLC-MS/MS metabolomic profiles from peel and flesh samples.The pink and blue-green shaded ellipses indicate the 95% confidence regions for peel and flesh samples, respectively. (**D**) Radar plots showing the relative abundance patterns of 11 metabolite classes in peel and flesh of each cultivar.Solid and dashed lines represent peel and flesh, respectively, while orange, red, and purple shaded areas indicate tissues assigned to the yellow, red, and purple color groups, respectively. (**E**) Violin plots comparing the relative abundance of each metabolite class across different cultivars in the peel and flesh. Dots from the peel and flesh of the same cultivar are connected by grey lines.Orange, red, and purple dots represent samples assigned to the yellow, red, and purple color groups, respectively.

A total of 248 metabolites belonging to 11 major classes were annotated by UPLC-MS/MS-based metabolomic analysis (Figure 3A,B; Table S2). Flavonoids accounted for the largest proportion, representing 37.50% of all identified metabolites, followed by phenolic acids, which accounted for 14.11%. The metabolite PCA separated peel from flesh. PC1 and PC2 accounted for 43.1% and 14.1% of the variance, respectively (Figure 3C). The clear separation indicated that tissue type was a major source of metabolic variation. Within the same tissue, samples also showed a degree of color-dependent clustering, and ‘FWMG’ was clearly separated from the other samples, suggesting that its purple peel and purple flesh possess a distinct metabolic composition.

Comparison of metabolite classes revealed clear tissue-specific patterns (Figure 3D,E). Flavonoids were predominant in the peel, particularly in purple-peel cultivars such as ‘FWMG’, ‘WH’, ‘WX’, and ‘XL01’. In contrast, the flesh showed stronger signals for amino acid derivatives, lipids and fatty acyls, nucleotide derivatives, organic acids, sugars and sugar alcohols, and terpenoids, with sugars and sugar alcohols being the most prominent. These patterns associate the peel with metabolites related to coloration and antioxidant capacity, whereas the flesh is more closely associated with metabolites contributing to flavor and nutritional quality.

### 3.4. Tissue-Specific Accumulation Patterns of Major Metabolite Classes

**Figure 4:**
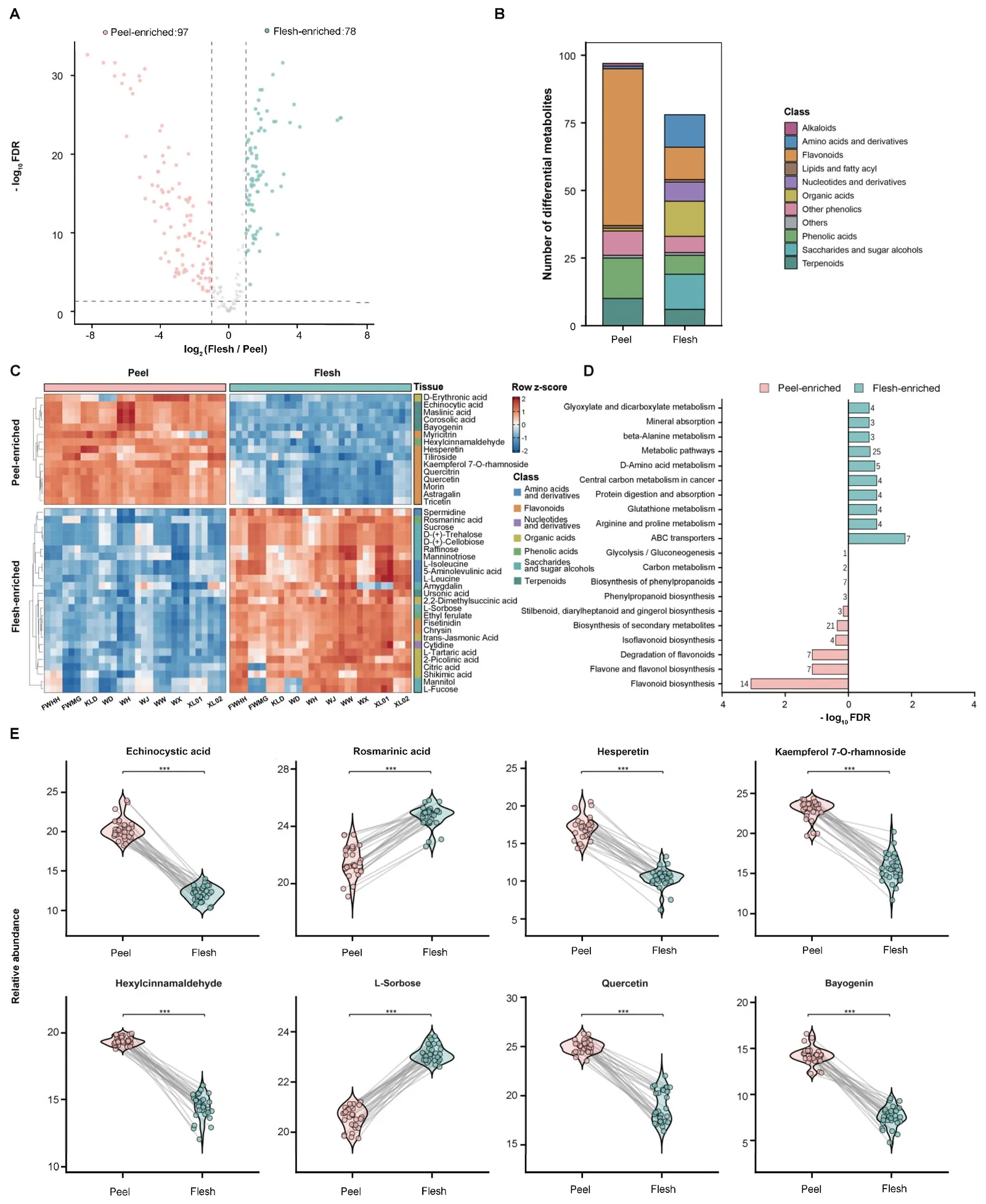
Tissue-specific accumulation of differentially accumulated metabolites between peel and flesh of *Prunus salicina* × *P. armeniaca* fruits. (**A**) Volcano plot showing peel- and flesh-enriched metabolites. (**B**) Chemical classification of peel- and flesh-enriched DAMs. The vertical dashed lines indicate log_2_(fold change) thresholds of −1 and 1, and the horizontal dashed line indicates an FDR threshold of 0.05. (**C**) Heatmap of the top 40 DAMs ranked by BH-adjusted *p* value from the limma analysis. Values were normalized by row Z-score transformation. (**D**) KEGG pathway enrichment analysis of peel- and flesh-enriched DAMs. Numbers beside bars indicate the number of mapped metabolites in each pathway. (**E**) Relative abundance of representative tissue-specific metabolites. Grey lines connect paired peel and flesh samples. Asterisks indicate significant differences (***, *p* < 0.001).

Differentially accumulated metabolites (DAMs) were then compared between peel and flesh to define tissue-related patterns. The analysis found 97 peel-enriched metabolites and 78 flesh-enriched metabolites (Figure 4A; Table S3). Among peel-enriched metabolites, flavonoids accounted for the largest proportion, followed by phenolic acids, other phenolics, and several terpenoids (Figure 4B). The heatmap of top-ranked DAMs showed clear tissue-specific accumulation patterns (Figure 4C; Table S4). Among these top-ranked tissue-associated DAMs, metabolites classified as peel-enriched included quercetin, quercitrin, myricitrin, hesperetin, kaempferol 7-O-rhamnoside, echinocystic acid, maslinic acid, and corosolic acid. In contrast, metabolites classified as flesh-enriched were mainly sugars, sugar alcohols, and organic acids, including sucrose, trehalose, cellobiose, raffinose, mannitol, L-sorbose, citric acid, shikimic acid, and tartaric acid.

Peel-enriched metabolites are mainly mapped to flavonoid, flavone and flavonol, and phenylpropanoid pathways. Flesh-enriched metabolites are mainly mapped to amino acid, carbon, and glutathione metabolism and to transport processes (Figure 4D). Comparisons of representative metabolites further supported these trends (Figure 4E). Overall, the peel preferentially accumulated secondary metabolites related to color formation, antioxidant activity, and defense, whereas the flesh preferentially accumulated primary metabolites associated with sugar–acid balance, flavor, and eating quality.

### 3.5. Analysis of Differentially Accumulated Metabolites Among Color Groups

**Figure 5:**
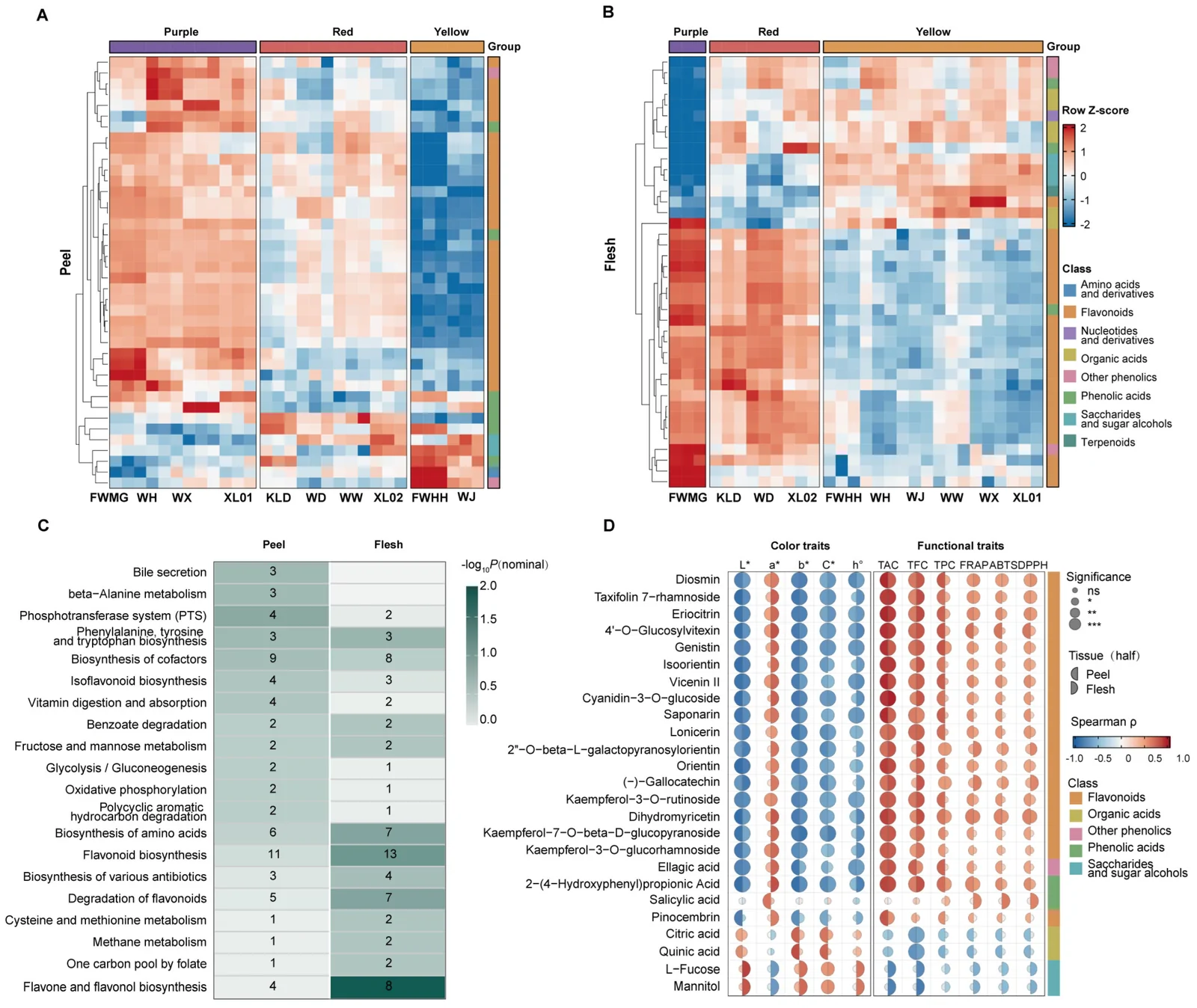
Color-related differentially accumulated metabolites in peel and flesh of *Prunus salicina* × *P. armeniaca* fruits. (**A**,**B**), Heatmaps of the top 40 color-associated metabolites in peel (**A**) and flesh (**B**), selected from the overall F-test of color groups and ranked by BH-adjusted *p* value. Values were normalized by row Z-score transformation. (**C**) KEGG pathway enrichment analysis. Numbers in each cell indicate the number of metabolites mapped to the corresponding pathway. (**D**) Spearman correlation analysis between representative color-related DAMs and fruit color, phytochemical and antioxidant traits.Significance was determined using BH-adjusted *p* values: ns, not significant (*p* ≥ 0.05); * *p* < 0.05; ** *p* < 0.01; and *** *p* < 0.001.

To elucidate the metabolic basis of color variation, pairwise comparisons among purple, red, and yellow groups were performed separately in peel and flesh. Purple and yellow tissues showed the largest DAM counts. This contrast included 76 DAMs in peel and 108 in flesh (Tables S5 and S7), which points to the largest metabolic gap between dark and light tissues (Figure S4A,B). Most pairwise comparisons contained more upregulated than downregulated metabolites. The heatmaps separated the color groups by their metabolite patterns. Flavonoids formed the largest share of the major DAMs (Figure 5A,B; Tables S6 and S8).

In peel, the color-related DAMs mapped mainly to phenylpropanoid and isoflavonoid biosynthesis, amino acid biosynthesis, and cofactor metabolism. In flesh, the main enriched pathways were flavone and flavonol biosynthesis, flavonoid biosynthesis, flavonoid degradation, and isoflavonoid biosynthesis (Figure 5C). These results suggest that flavonoid- and phenylpropanoid-related metabolic pathways are important metabolic bases underlying color differences in both peel and flesh.

Most flavonoid-related DAMs were positively associated with a*, TAC, and TFC and negatively associated with L*, b*, C*, and *h*°, consistent with enrichment in red or purple tissues (Figure 5D). Their correlations with TPC and antioxidant indicators were generally positive, but the strength and significance of these relationships were tissue-dependent. Cyanidin-3-O-glucoside had the most stable link with tissue coloration and TAC, especially in flesh. Dihydromyricetin, taxifolin 7-rhamnoside, eriocitrin, genistin, and several flavonoid glycosides were connected to phytochemical and antioxidant traits in a broader, but less tissue-uniform manner. These DAMs were therefore carried forward for integration with color-gradient and machine-learning analyses.

### 3.6. Machine Learning-Assisted Identification of Discriminative and Predictive Metabolic Features

Random forest analysis was used as an additional test of whether metabolite abundance patterns could separate tissues, color groups, and antioxidant capacity. The peel-flesh classifier achieved an AUC of 1.000 (Figure S5A), matching the strong tissue separation observed in the metabolomic PCA. Separate peel and flesh color models also separated purple, red, and yellow samples, as reflected by the normalized confusion matrices (Figure S5B,C). These models support the presence of tissue-specific metabolomic signatures for fruit color rather than a single shared color profile.

**Figure 6:**
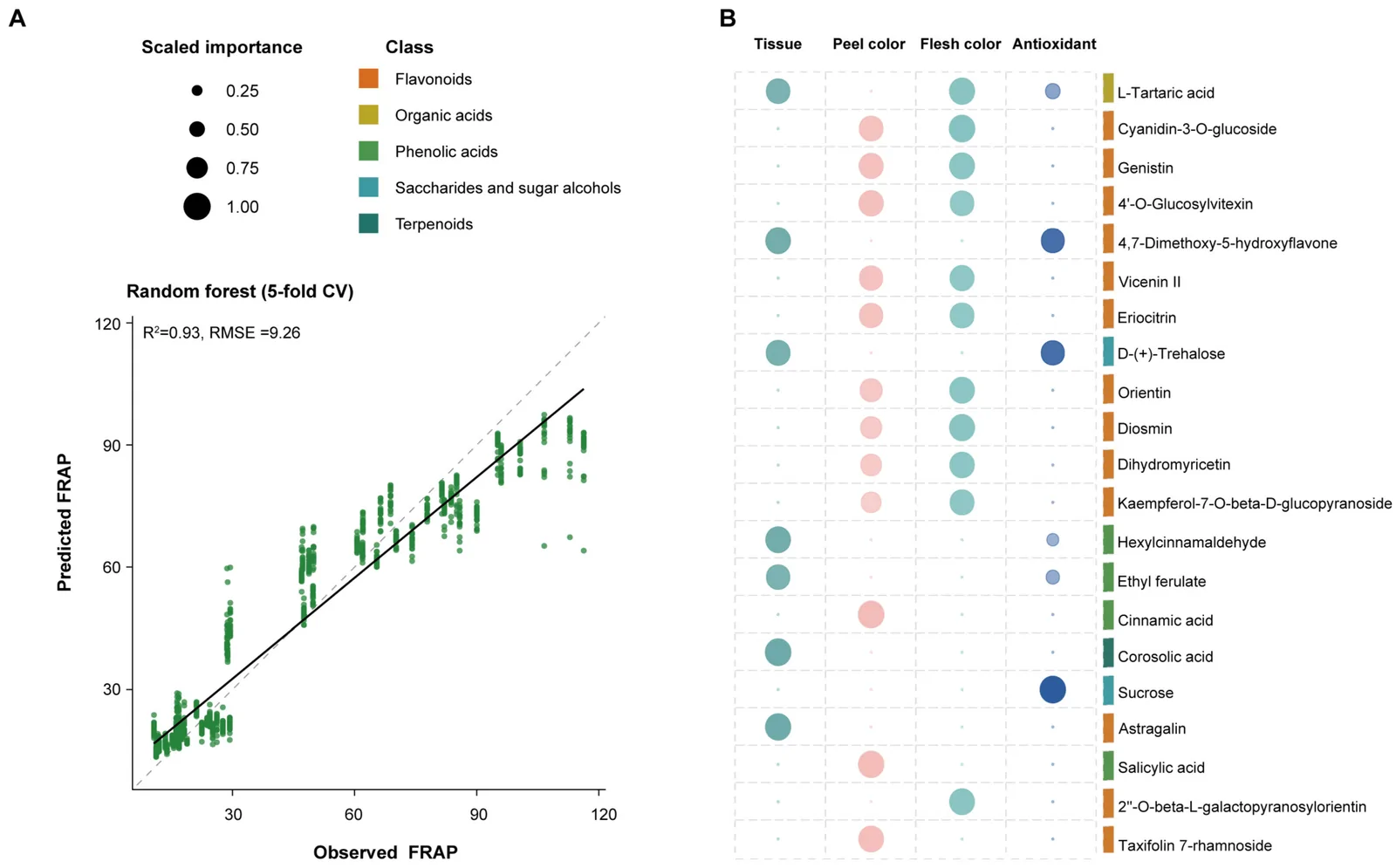
Random forest-assisted identification of predictive features associated with tissue type, fruit color and antioxidant activity in *Prunus salicina* × *P. armeniaca* fruits. (**A**) Observed versus predicted FRAP values using the random forest regression model. The dashed line indicates the 1:1 reference line, and the solid line indicates the fitted regression line. (**B**) Importance scores for selected metabolites in tissue classification, peel-color classification, flesh-color classification, and FRAP prediction. Bubble size represents the scaled importance score. The side bars identify metabolite classes. FRAP, ferric reducing antioxidant power; RMSE, root mean square error.

With FRAP as the response variable, random forest regression produced cross-validated R^2^ = 0.93 and RMSE = 9.26 (Figure 6A), indicating that the measured metabolite profiles captured much of the variation in reducing capacity. The variable-importance profiles differed by task (Figure 6B). Tissue classification was supported by metabolites such as corosolic acid, astragalin, 4,7-dimethoxy-5-hydroxyflavone, ribitol, shikimic acid, echinocystic acid, and kaempferol 7-O-rhamnoside. Peel color classification was associated mainly with phenolic acids and flavonoids, including cinnamic acid, salicylic acid, taxifolin 7-rhamnoside, 4′-O-glucosylvitexin, genistin, and cyanidin-3-O-glucoside. Flesh color classification drew on a wider mixture of flavonoids and primary metabolites, with cyanidin-3-O-glucoside, L-tartaric acid, diosmin, genistin, orientin, mannitol, L-fucose, and dihydromyricetin among the leading features. For FRAP prediction, high-ranking features included sucrose, D-(+)-trehalose, 4,7-dimethoxy-5-hydroxyflavone, D-(+)-cellobiose, chrysin, and L-tartaric acid. The three modeling tasks therefore shared some signals but emphasized different parts of the metabolome.

### 3.7. Integrated Screening of Metabolites Associated with Color Variation and Antioxidant Capacity

**Figure 7:**
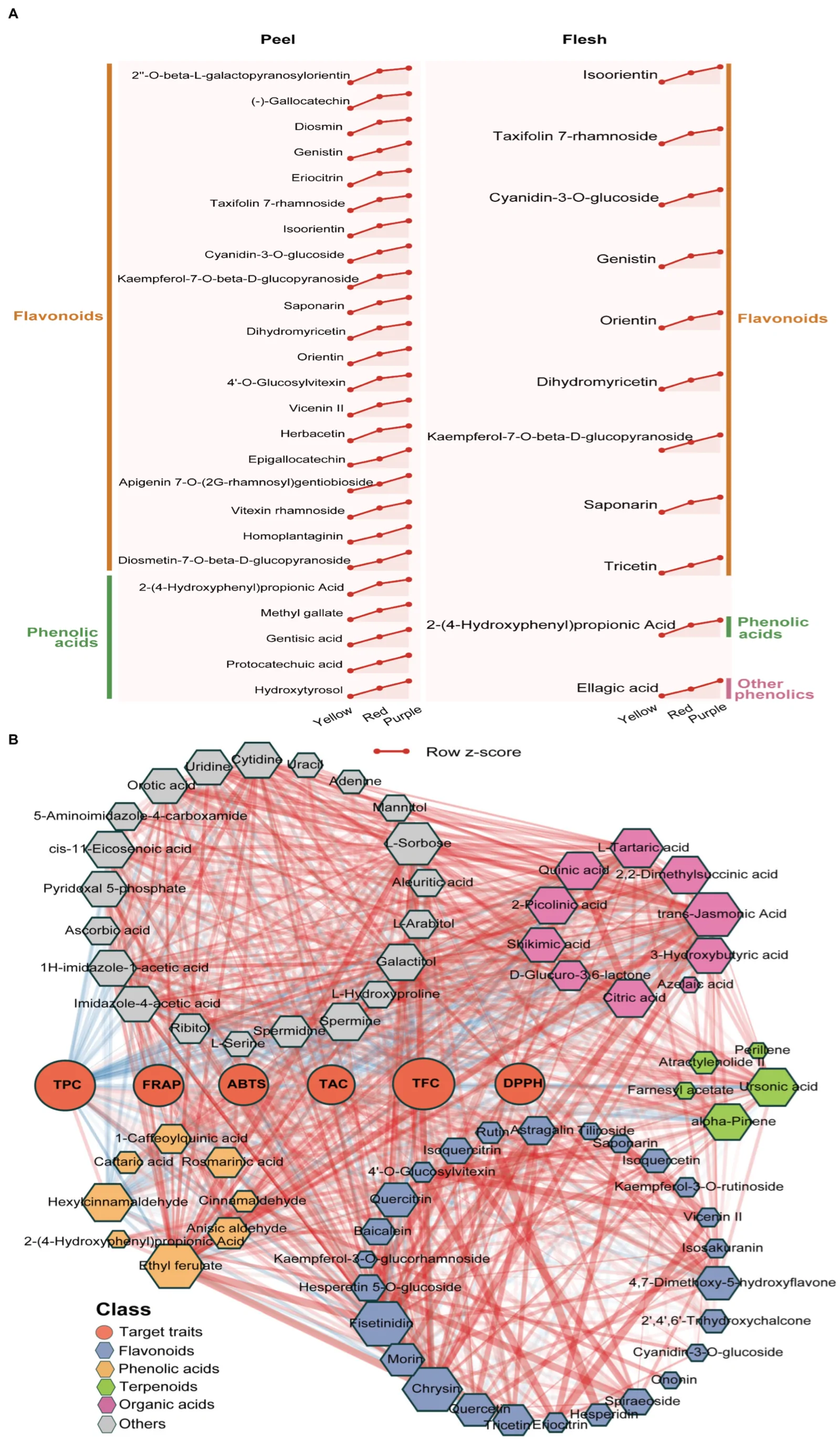
Integrated screening of candidate metabolites associated with fruit color and antioxidant potential in *Prunus salicina* × *P. armeniaca* peel and flesh. (**A**) Metabolites showing progressive accumulation along the color gradient in peel and flesh. Values were normalized by row Z-score transformation. (**B**) Phenotype-anchored metabolite–trait correlation network. Node colors indicate metabolite classes or target traits. Red and blue edges represent positive and negative correlations, respectively.

Based on these positive associations (Figure 5D), the integrated analysis combined color-gradient metabolites with a metabolite-trait network anchored by phytochemical and antioxidant traits (Figure 7). Metabolites that gradually accumulated with deeper peel and flesh coloration were mainly flavonoids, phenolic acids, and other phenolic compounds (Figure 7A). These metabolites largely overlapped with the previously identified color-related DAMs, trait-associated metabolites, and machine learning-prioritized metabolites, suggesting that they may jointly contribute to color variation and functional quality formation.

Using thresholds of |ρ| ≥ 0.70 and BH-adjusted *p* < 0.05, the functional trait-anchored network identified six functional trait nodes and 70 metabolite nodes (Figure 7B). Among them, TFC and TPC were the most highly connected phenotypic anchors, being associated with 63 and 61 metabolites, respectively. DPPH, ABTS, and FRAP showed a highly overlapping set of positively correlated metabolites. Quercitrin exhibited the strongest positive association with each of the three antioxidant assays, followed by tiliroside, while 4′-O-glucosylvitexin, isoquercitrin, and spiraeoside were also strongly and consistently associated with all three assays. TAC was significantly correlated with 43 metabolites, including 4′-O-glucosylvitexin, eriocitrin, vicenin II, kaempferol-3-O-rutinoside, saponarin, and cyanidin-3-O-glucoside. Cyanidin-3-O-glucoside was the only anthocyanin annotated in this dataset. Its abundance was positively related to TAC. Meanwhile, several non-anthocyanin flavonoid glycosides were also closely associated with TAC and antioxidant indicators. Among all candidate metabolites, 4′-O-glucosylvitexin showed the strongest association with TAC and was also positively correlated with TPC, TFC, DPPH, ABTS, and FRAP, suggesting its potential functional importance.

Across tissue-specific correlations, random forest color classification, and color-gradient analysis, cyanidin-3-O-glucoside, dihydromyricetin, genistin, and orientin showed the most consistent cross-tissue support for color association. Taxifolin 7-rhamnoside, isoorientin, eriocitrin, vicenin II, 4′-O-glucosylvitexin, and diosmin also showed color-related evidence, but with stronger tissue-dependence. Cyanidin-3-O-glucoside, taxifolin 7-rhamnoside, dihydromyricetin, and vicenin II were selected for detailed visualization because they were supported by multiple analyses and represented distinct flavonoid subclasses and accumulation patterns, including an anthocyanin, dihydroflavonols, and a flavone C-glycoside. Visualization of representative metabolites showed that cyanidin-3-O-glucoside, taxifolin 7-rhamnoside, and dihydromyricetin increased significantly with deeper coloration in both peel and flesh (Figure S6). Vicenin II showed a similar increasing pattern in the peel. In flesh, vicenin II was more abundant in red and purple tissues than in yellow tissues. The red and purple groups did not differ significantly. These results suggest that fruit color variation and functional quality formation are associated with a coordinated metabolic network involving anthocyanins, dihydroflavonols, flavone glycosides, flavonol glycosides, and phenolic acids.

### 3.8. CNQI-Based Evaluation of Nutritional Quality

**Figure 8:**
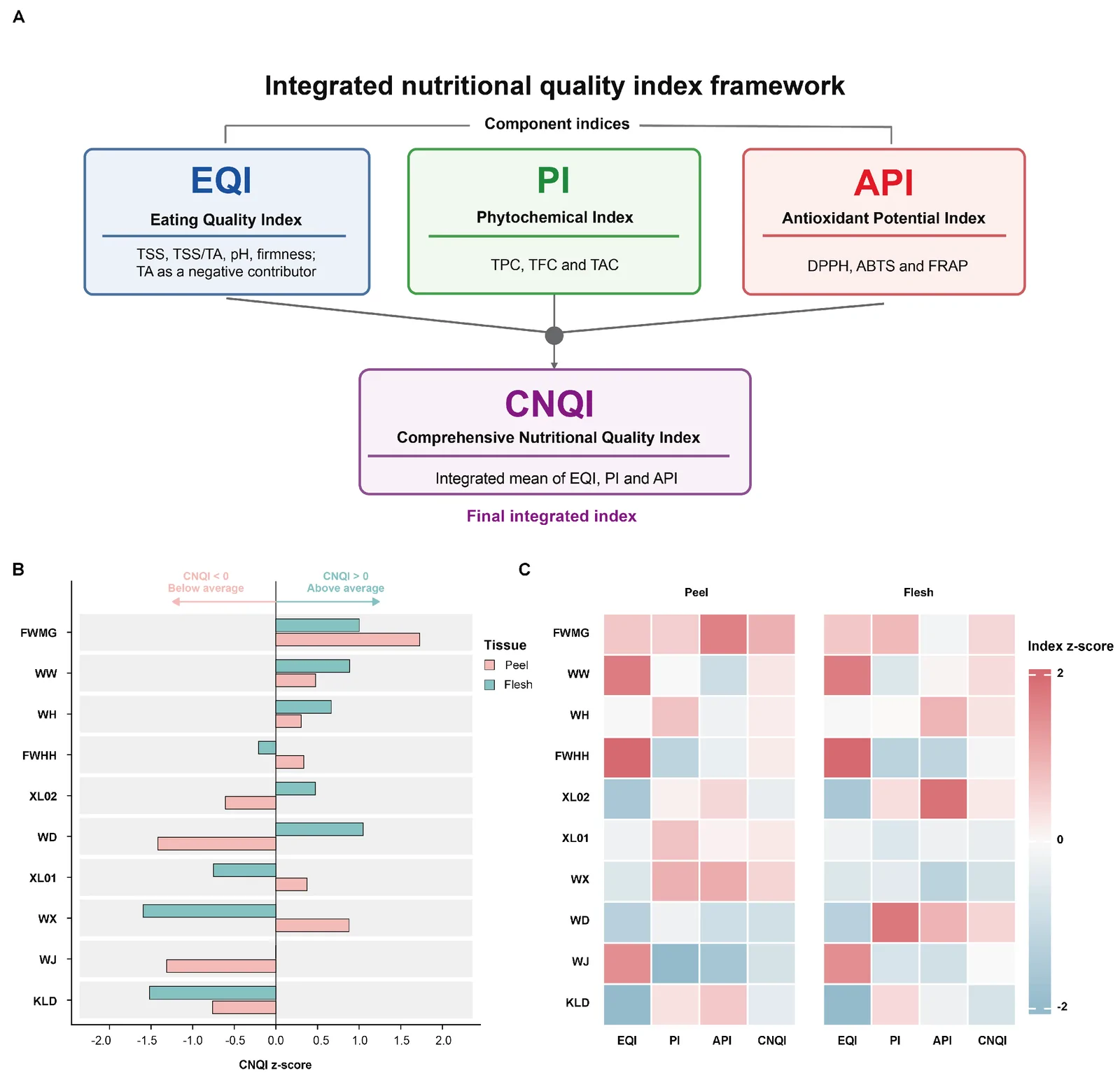
Integrated comprehensive nutritional quality evaluation of *Prunus salicina* × *P. armeniaca* peel and flesh. (**A**) Framework of the CNQI. (**B**) CNQI z-score ranking of peel and flesh among the 10 cultivars. Positive and negative values indicate above- and below-average comprehensive nutritional quality within the same tissue, respectively. (**C**) Heatmap showing the z-score profiles of EQI, PI, API and CNQI.

CNQI compared cultivars after combining eating quality, bioactive compound accumulation, and antioxidant capacity (Figure 8A). The index incorporated EQI, PI, and API. CNQI z-scores separated cultivars by their peel and flesh quality profiles (Figure 8B). For peel, ‘FWMG’ ranked first, followed by ‘WX’ and ‘WW’; ‘XL02’, ‘KLD’, ‘WJ’, and ‘WD’ were below the tissue mean. For flesh, ‘WD’ had the highest score, and ‘FWMG’, ‘WW’, ‘WH’, and ‘XL02’ also had positive CNQI values.

The heatmap indicated why these rankings differed among tissues (Figure 8C). Peel of ‘FWMG’ had high EQI, PI, and API values. Peel of ‘WX’ was favored mainly by PI and API, while peel of ‘WW’ was favored by EQI. In flesh, ‘FWMG’ had higher EQI and PI, whereas ‘WH’ and ‘XL02’ had higher API. The high flesh CNQI of ‘WD’ was driven by PI and API despite its lower EQI. Together, the rankings separate a balanced profile in ‘FWMG’ from the flesh-oriented profile of ‘WD’ and the peel-oriented functional profile of ‘WX’.

## 4. Discussion

### 4.1. Peel- and Flesh-Specific Bioactive Compounds and Antioxidant Capacity

Peel and flesh differed clearly in their food-quality chemistry. In these hybrids, peel had higher TPC, TFC, TAC, DPPH, ABTS, and FRAP values than flesh, indicating that phenolic and flavonoid accumulation was concentrated in the outer tissue. This pattern is consistent with reports in Prunus fruit, where plum peel contained more anthocyanins, flavonols, and phenolic acids than flesh [25,26]. Peach and nectarine also contain more peel phenolics and show stronger peel antioxidant activity than pulp [27,28]. Phenolic enrichment in peel may therefore reflect the protective function of the fruit surface rather than a cultivar-specific exception.

The metabolite profiles showed the same tissue difference. Flavonoids formed the largest group among the 248 annotated metabolites. The peel-enriched DAMs mapped mainly to flavonoid, flavone and flavonol, isoflavonoid, and phenylpropanoid pathways. These data point to stronger phenylpropanoid-flavonoid activity in peel. European plum peel also contains many phenylpropanoid and flavonoid compounds [25]. Light, physical stress, and pathogen exposure at the fruit surface may promote these defensive phenolics [26,29]. A recent untargeted metabolomic study of *P. salicina* likewise demonstrated distinct phytochemical distributions between the peel and pulp [30].

The relationship between phytochemical composition and antioxidant capacity was tissue-dependent. In the peel, darker coloration coincided with higher TPC, TFC, TAC, DPPH, ABTS, and FRAP values, indicating that peel antioxidant activity was associated with the combined accumulation of anthocyanins and other phenolics. Similar relationships between phenolic accumulation and antioxidant activity have been reported in plum and red-fleshed apple [29,31]. More recently, cultivar-dependent variation in phenolic composition, anthocyanin content, and antioxidant capacity was also reported among 11 *Prunus salicina* cultivars [32]. In the flesh, however, the red-fleshed cultivars showed the highest antioxidant capacity despite not consistently having the highest total phytochemical contents, suggesting a greater influence of individual antioxidant compounds. Indeed, Venter et al. [33] reported that total anthocyanin content in red-fleshed plum was weakly associated with some antioxidant assays, whereas flavan-3-ols showed stronger associations. In the present study, quercitrin, tiliroside, 4′-O-glucosylvitexin, isoquercitrin, and spiraeoside were among the phenolic metabolites most strongly and consistently associated with DPPH, ABTS, and FRAP. Their shared associations suggest that multiple flavonoid glycosides may collectively contribute to antioxidant potential. Nevertheless, differences in radical structure, reaction medium, and electron- or hydrogen-transfer mechanisms can result in assay-specific responses to phenolic compounds [34]. Because the present evidence is correlative, these metabolites should be considered candidate contributors rather than compounds proven to be directly responsible for antioxidant activity.

### 4.2. Color Variation Reflects Coordinated Changes in Flavonoid-Related Metabolism

Fruit color variation usually reflects coordinated changes in secondary metabolism rather than the accumulation of a single pigment. Although anthocyanins are the most direct contributors to red or purple coloration, flavonols, flavonoid glycosides, phenolic acids, and other phenolics often change together with anthocyanins [35,36]. In this study, the largest numbers of DAMs were detected between purple and yellow tissues in both peel and flesh, indicating the strongest metabolic differentiation between dark- and light-colored tissues. The KEGG results placed most color-related DAMs in flavonoid biosynthesis, flavone and flavonol biosynthesis, flavonoid degradation, isoflavonoid biosynthesis, and related phenylpropanoid pathways. Consistent with these findings, a recent tissue-resolved study of Japanese plum also revealed cultivar- and tissue-dependent differences in anthocyanin accumulation between the peel and flesh [10]. This pattern is consistent with studies in other colored fruits. Studies of red-fleshed apple and peach also found changes in non-anthocyanin flavonoids and phenolic acids alongside anthocyanin accumulation [12,31]. Wampee peel shows a similar coordinated shift in flavonoid metabolism during color change [37]. During fruit maturation, coordinated activation and redirection of phenylpropanoid-flavonoid metabolism can generate structurally diverse compounds, including anthocyanins, flavonols, flavone glycosides, phenolic acids, and their conjugated derivatives. Glycosylation and other structural modifications may influence anthocyanin stability and accumulation, while non-covalent copigmentation with flavonoids and phenolic acids can further modify color intensity, stability, and hue [38,39]. In the present mature-fruit panel, these related metabolites generally increased from yellow to red and purple tissues. Cyanidin derivatives may contribute directly to red or purple coloration, whereas other flavonoids and phenolic compounds may influence the observed phenotype through shared pathway flux, antioxidant-related functions, or copigmentation. Therefore, coloration in these plum–apricot hybrids is better interpreted as the outcome of coordinated remodeling across multiple branches of the phenylpropanoid-flavonoid network rather than as a simple increase in a single anthocyanin end product.

### 4.3. Candidate Metabolites Associated with Coloration and Antioxidant Potential

The different analyses identified related, but not identical, sets of metabolites for color, phytochemical content, and antioxidant capacity. Three analyses repeatedly linked cyanidin-3-O-glucoside, dihydromyricetin, genistin, and orientin with color in both tissues. These analyses were tissue-specific correlations, random forest classification, and color-gradient screening. Taxifolin 7-rhamnoside, isoorientin, eriocitrin, vicenin II, 4′-O-glucosylvitexin, and diosmin also showed color links. Their evidence was stronger in one tissue than in the other. Some metabolites may therefore provide shared color signals across peel and flesh. Others appear to reflect tissue-specific metabolism.

Among the metabolites in our study, cyanidin-3-O-glucoside was the only anthocyanin that met the detection and annotation criteria of the current UPLC-MS/MS platform. It showed the clearest association with red or purple coloration and TAC. Previous studies have identified cyanidin-3-O-glucoside and cyanidin-3-O-rutinoside as the predominant anthocyanins in cultivated plum fruits [40,41]. The absence of these anthocyanins, including 3,5-O-diglucoside anthocyanins, cyanidin-3-O-rutinoside, and malvidin-3-O-glucoside, were confidently annotated in the present dataset. However, its absence from the final data matrix does not exclude its possible presence below the detection or annotation threshold. Cyanidin-3-O-glucoside may therefore contribute to the coloration of *Prunus salicina* × *P. armeniaca* fruits, but it should not be regarded as the only anthocyanin present or as fully explaining the observed color variation. Dihydromyricetin and taxifolin 7-rhamnoside are associated with the dihydroflavonol branch upstream of anthocyanin and flavonol biosynthesis. Flavonol synthase and dihydroflavonol 4-reductase compete for the same substrates. This competition can direct flux toward different flavonoid branches [42]. Their accumulation may therefore reflect changes in upstream flavonoid flux accompanying tissue coloration [37]. Some flavonoid glycosides may also contribute to coloration. Orientin has been shown to enhance anthocyanin color intensity in a strawberry model solution [38], whereas taxifolin has been reported to exert a copigmentation effect with cyanidin-3-O-glucoside [39].

The trait-based network did not rank all color candidates in the same way. Cyanidin-3-O-glucoside was linked mainly with TAC, whereas 4′-O-glucosylvitexin had the largest number of network connections and was positively associated with TPC, TFC, TAC, DPPH, ABTS, and FRAP. Eriocitrin and vicenin II were also connected with phytochemical content and antioxidant traits. Thus, 4′-O-glucosylvitexin appears to bridge broad flavonoid accumulation and antioxidant activity, while cyanidin-3-O-glucoside is more closely tied to anthocyanin content and color. Published antioxidant evidence for diosmin, eriocitrin, and vicenin II is consistent with possible functional roles for these compounds [43,44]. Taken together, the data show that color, phytochemical content, and antioxidant activity are related but separate traits. Each trait was represented by a partly shared metabolite set.

### 4.4. Integrated Metabolomic Screening and Quality Evaluation

Random forest analysis provided a complementary approach for prioritizing metabolites associated with tissue type, color phenotype, and antioxidant capacity. Within the present dataset, the models effectively distinguished peel from flesh and separated the different color groups, further demonstrating that tissue type and color phenotype are characterized by distinct metabolomic profiles. A recent study similarly combined metabolite profiling with unsupervised machine learning to differentiate 12 Japanese plum cultivars and demonstrated that both cultivar and postharvest stage contributed to variation in their metabolic profiles [45]. Similar machine-learning strategies have been applied to classify volatile metabolites in pigmented onions [46], identify metabolites associated with color, taste, and flavor in blueberries [47], and screen candidate biomarkers of postharvest quality in atemoya [48]. In the present study, several high-importance color-classification features were also supported by DAM analysis, color-associated accumulation patterns, or trait correlations. This agreement across complementary analytical approaches strengthens their relevance as candidate metabolites associated with fruit color and functional quality, although their biological roles require further validation.

CNQI added a cultivar-level view by bringing eating quality, phytochemical accumulation, and antioxidant potential into one comparison. Recent comparisons among five *Prunus salicina* cultivars from Sichuan likewise revealed substantial cultivar-dependent variation in physicochemical attributes, antioxidant capacity, and volatile profiles [49]. Composite indices have also been used in fruit-quality studies of kiwifruit and strawberry [50,51]. In our dataset, ‘FWMG’ performed well in both peel and flesh, ‘WD’ ranked highest for flesh, and ‘WX’ derived much of its advantage from peel phytochemicals and antioxidant activity. EQI captured whole-fruit eating traits, whereas PI and API described tissue-specific functional traits. Their combination allowed cultivar ranking to retain both forms of quality information.

Equal weighting was chosen because there was no biological or agronomic basis for weighing EQI, PI, and API differently in this dataset. It also avoided tuning the index to a limited number of cultivars. CNQI should therefore be interpreted as a transparent comparison metric, not as an optimized prediction model. Random forest analysis and CNQI served different purposes: the former prioritized metabolites associated with traits, and the latter ranked cultivars by combined quality. Together, they connect metabolite-level screening with practical evaluation of plum–apricot hybrid fruit.

## 5. Conclusions

This study demonstrates that tissue type is a major source of metabolic variation among the ten *Prunus salicina* × *P. armeniaca* cultivars. The peel was enriched in phenolic compounds, particularly flavonoids, and showed stronger antioxidant capacity, whereas the flesh contained higher relative abundances of sugars, sugar alcohols, organic acids, and other metabolites associated with flavor and eating quality. Fruit color variation was mainly associated with coordinated changes in phenylpropanoid and flavonoid metabolism, with the largest metabolic differences observed between purple and yellow tissues. Integration of tissue-specific correlations, random forest models, and color-gradient analysis identified cyanidin-3-O-glucoside, dihydromyricetin, genistin, and orientin as candidate metabolites associated with color variation. Cyanidin-3-O-glucoside showed the most consistent association with tissue color and anthocyanin content, whereas 4′-O-glucosylvitexin was broadly associated with phytochemical and antioxidant traits. The CNQI analysis further revealed complementary cultivar- and tissue-specific quality profiles, highlighting the importance of evaluating peel and flesh separately. Overall, these findings provide a tissue-resolved basis for the evaluation and utilization of plum–apricot hybrid germplasm, although targeted quantification and functional validation are required to confirm the roles of the identified candidate metabolites.

## Data Availability

The original contributions presented in this study are included in the article/Supplementary Materials. Further inquiries can be directed to the corresponding author.

## Supplementary Materials

The following supporting information can be downloaded at https://www.mdpi.com/article/10.3390/foods15162790/s1, Figure S1. Fruit physicochemical quality, color characteristics, phytochemical contents, and antioxidant capacities of ten *Prunus salicina* × *P. armeniaca* cultivars. (A) Total soluble solids (TSS); (B) titratable acidity (TA); (C) TSS/TA ratio; (D) pH; (E) firmness; (F) fruit weight; (G) longitudinal diameter; (H) transverse diameter; (I) fruit shape index; (J–N) CIELAB color parameters, including *L**, *a**, *b**, *C**, and *h*°; (O) total phenolic content (TPC); (P) total flavonoid content (TFC); (Q) total anthocyanin content (TAC); and (R–T) DPPH radical-scavenging activity, ABTS radical-scavenging activity, and ferric reducing antioxidant power (FRAP), respectively. Values are presented as means ± standard deviation of three biological replicates; Figure S2. Spearman correlation matrix of fruit physicochemical quality, peel color, phytochemical, and antioxidant traits in ten *Prunus salicina* × *P. armeniaca* cultivars. Correlation coefficients are shown in the upper triangle, variable distributions along the diagonal, and pairwise scatterplots in the lower triangle. Asterisks indicate significant correlations (*, *p* < 0.05; **, *p* < 0.01; ***, *p* < 0.001); Figure S3. Spearman correlation matrix of fruit physicochemical quality, flesh color, phytochemical, and antioxidant traits in ten *Prunus salicina* × *P. armeniaca* cultivars. Correlation coefficients are shown in the upper triangle, variable distributions along the diagonal, and pairwise scatterplots in the lower triangle. Asterisks indicate significant correlations (*, *p* < 0.05; **, *p* < 0.01; ***, *p* < 0.001); Figure S4. (A,B), Distribution of up- and down-regulated differentially accumulated metabolites (DAMs) in pairwise comparisons among purple, red and yellow color groups in peel (A) and flesh (B). Red and green points indicate up- and down-regulated DAMs, respectively, and grey points indicate non-significant metabolites; Figure S5. Random forest-assisted identification of predictive features associated with tissue type, fruit color and antioxidant activity in *Prunus salicina* × *P. armeniaca* fruits. (A) Receiver operating characteristic (ROC) curve for peel-flesh classification based on metabolomic profiles. (B,C), Normalized confusion matrices of random forest models for color classification in peel (B) and flesh (C) using repeated group-based 5-fold cross-validation with 20 repeats. RF, random forest; CV, cross-validation; AUC, area under the curve; Figure S6. Relative abundance of representative candidate metabolites in peel and flesh across different groups. Asterisks indicate significant differences (***, *p* < 0.001); ns, not significant. Table S1. Days after full bloom (DAFB) at which different cultivars were sampled at maturity; Table S2. Relative abundances of all metabolites annotated in the peel and flesh samples of ten plum–apricot hybrid cultivars using widely targeted UPLC–MS/MS analysis; Table S3. All DAMs identified between peel and flesh of *Prunus salicina* × *P. armeniaca* fruits; Table S4. Top 40 DAMs between peel and flesh of *Prunus salicina* × *P. armeniaca* fruits; Table S5. All DAMs identified among purple, red, and yellow peel groups of *Prunus salicina* × *P. armeniaca* fruits; Table S6. Top 40 color-associated DAMs in the peel of *Prunus salicina* × *P. armeniaca* fruits; Table S7. All DAMs identified among purple, red, and yellow flesh groups of *Prunus salicina* × *P. armeniaca* fruits; Table S8. Top 40 DAMs among different color groups in the flesh of *Prunus salicina* × *P. armeniaca* fruits.
