# Supplementary material for: Tissue-Resolved Metabolomic Profiling of Ten Prunus salicina × P. armeniaca Cultivars Reveals Peel–Flesh Metabolic Differences Associated with Fruit Color and Antioxidant Capacity
Source: Foods. 2026 Aug 9;15(16):2790. doi: 10.3390/foods15162790 (PMC13512046; doi:10.3390/foods15162790)
Supplement: Supplementary file 1 [file foods-15-02790-s001.zip › Figure S1.pdf]

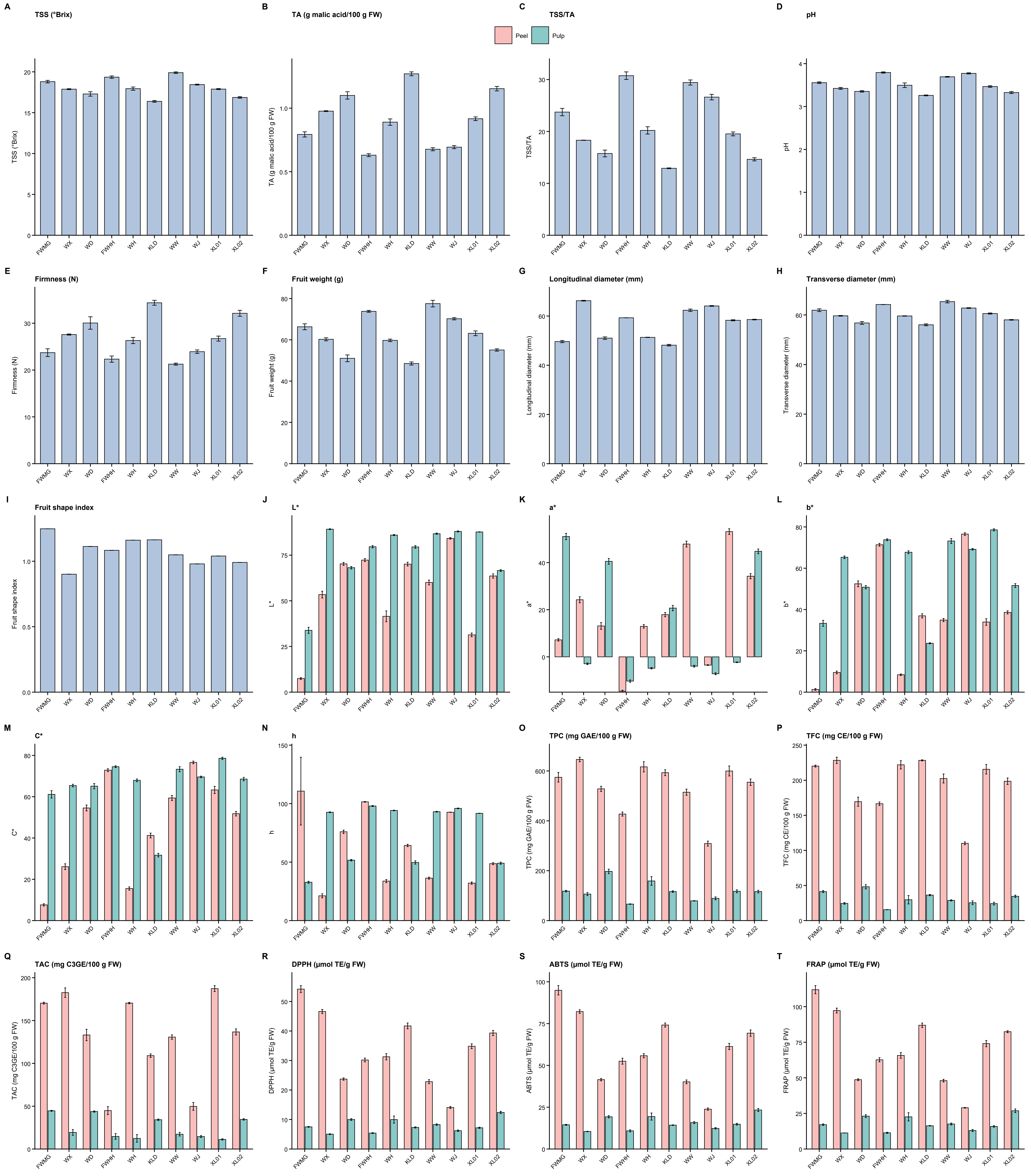

A
TSS (°Brix)
B
TA (g malic acid/100 g FW)
C
TSS/TA
D
pH
Peel
Pulp
E
Firmness (N)
F
Fruit weight (g)
G
Longitudinal diameter (mm)
H
Transverse diameter (mm)
I
Fruit shape index
J
L*
K
a*
L
b*
M
C*
N
h
O
TPC (mg GAE/100 g FW)
P
TFC (mg CE/100 g FW)
Q
TAC (mg C3GE/100 g FW)
R
DPPH (μmol TE/g FW)
S
ABTS (μmol TE/g FW)
T
FRAP (μmol TE/g FW)
FWMG
WX
WD
FWHH
WH
KLD
WW
WJ
XL01
XL02
