# Supplementary material for: Tissue-Resolved Metabolomic Profiling of Ten Prunus salicina × P. armeniaca Cultivars Reveals Peel–Flesh Metabolic Differences Associated with Fruit Color and Antioxidant Capacity
Source: Foods. 2026 Aug 9;15(16):2790. doi: 10.3390/foods15162790 (PMC13512046; doi:10.3390/foods15162790)

Pulp phenotype correlations (n = 30, Spearman)

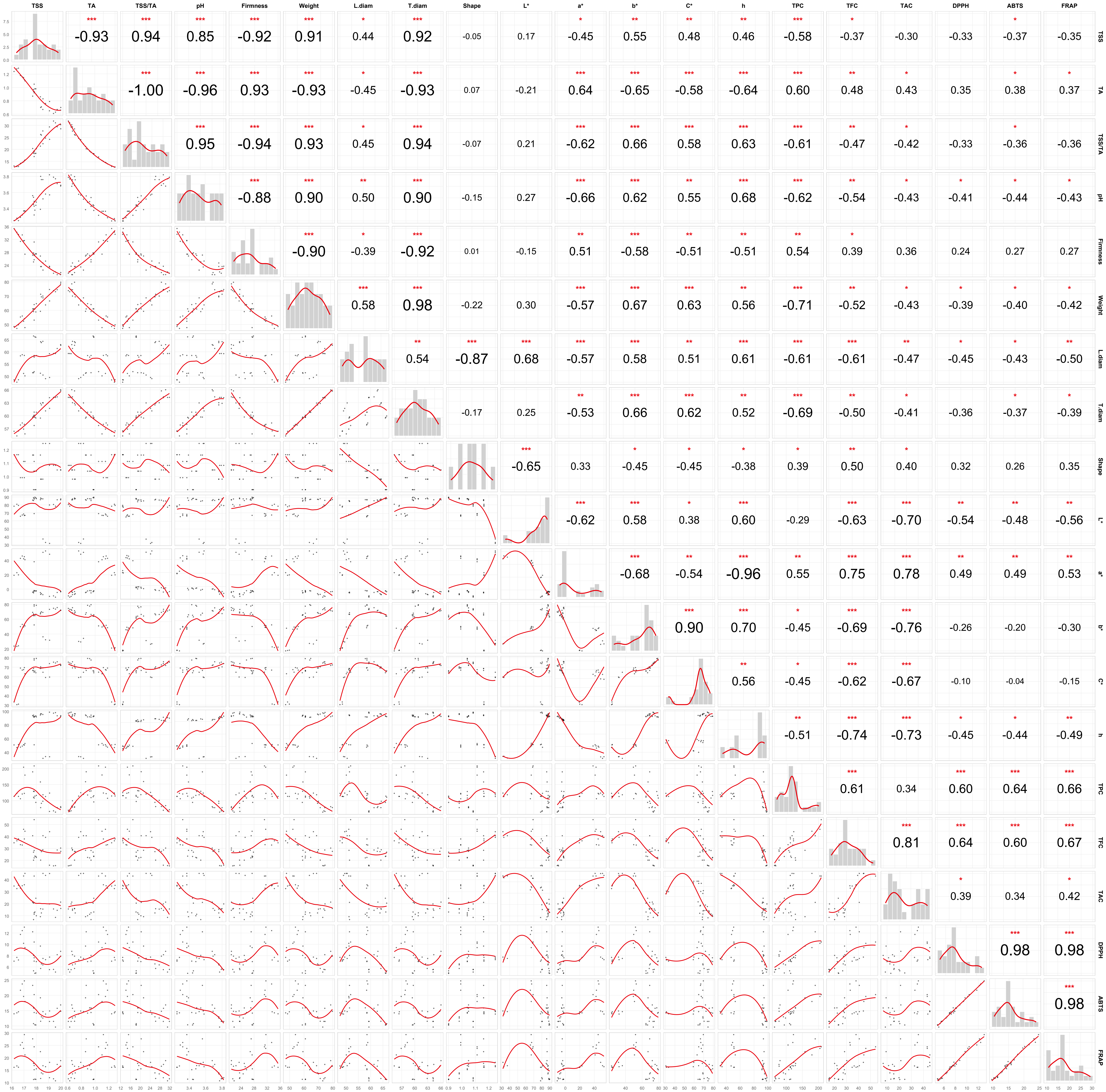

Supplement: Supplementary file 1 [file foods-15-02790-s001.zip › Figure S3.pdf]
