# Supplementary material for: Tissue-Resolved Metabolomic Profiling of Ten Prunus salicina × P. armeniaca Cultivars Reveals Peel–Flesh Metabolic Differences Associated with Fruit Color and Antioxidant Capacity
Source: Foods. 2026 Aug 9;15(16):2790. doi: 10.3390/foods15162790 (PMC13512046; doi:10.3390/foods15162790)
Supplement: Supplementary file 1 [file foods-15-02790-s001.zip › Figure S4.pdf]

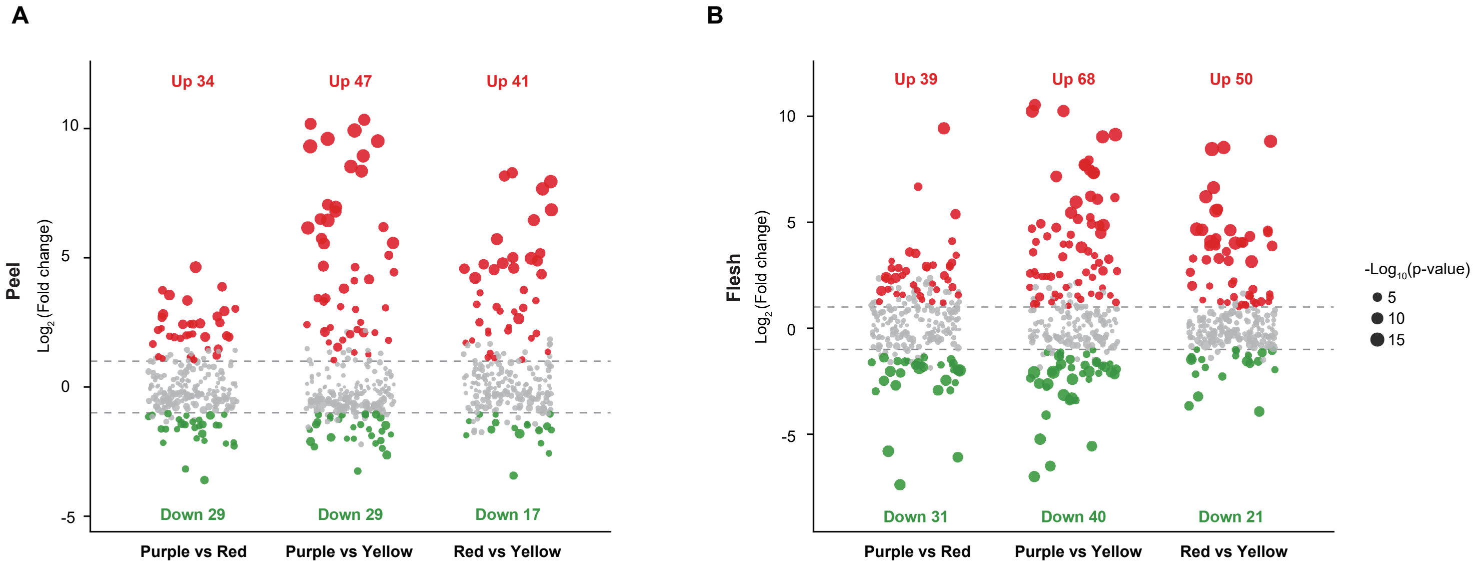
A
B
Up 34
Up 47
Up 41
Up 39
Up 68
Up 50
Peel
Log2 (Fold change)
Flesh
10
5
0
-5
Down 29
Down 29
Down 17
Down 31
Down 40
Down 21
Purple vs Red
Purple vs Yellow
Red vs Yellow
-Log10(p-value)
15
