# Supplementary material for: Tissue-Resolved Metabolomic Profiling of Ten Prunus salicina × P. armeniaca Cultivars Reveals Peel–Flesh Metabolic Differences Associated with Fruit Color and Antioxidant Capacity
Source: Foods. 2026 Aug 9;15(16):2790. doi: 10.3390/foods15162790 (PMC13512046; doi:10.3390/foods15162790)
Supplement: Supplementary file 1 [file foods-15-02790-s001.zip › Figure S5.pdf]

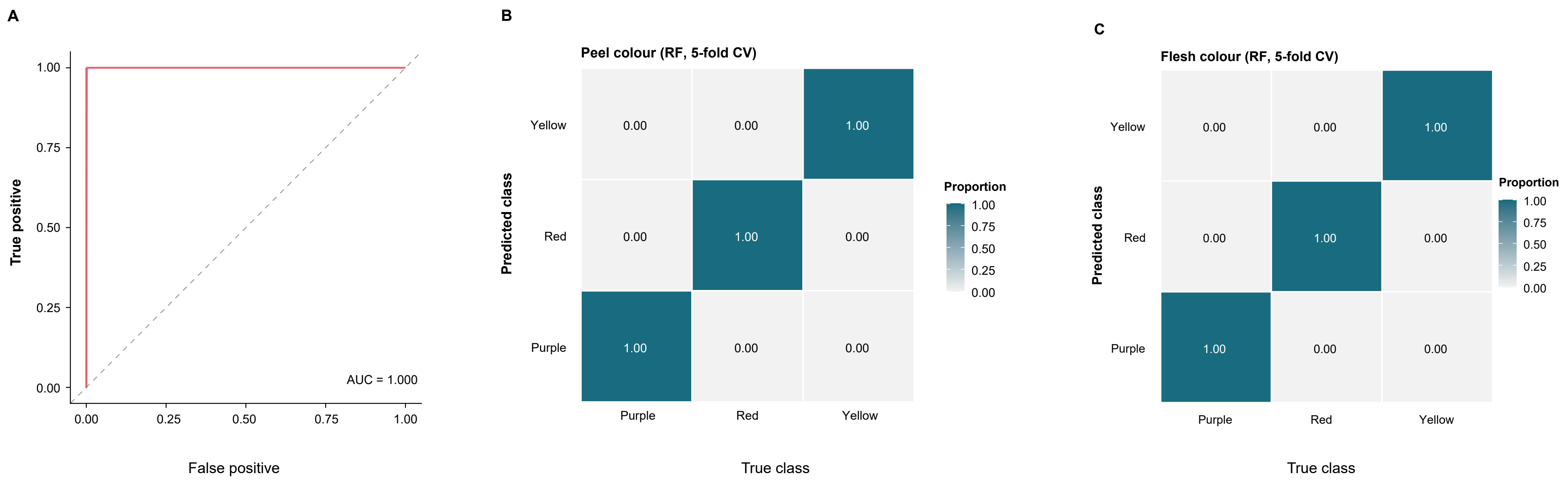

A
1.00
0.75
0.50
0.25
0.00
True positive
0.00
0.25
0.50
0.75
1.00
AUC = 1.000
False positive
B
Peel colour (RF, 5-fold CV)
Predicted class
Yellow
Red
Purple
0.00
0.00
1.00
0.00
1.00
0.00
1.00
0.00
0.00
Purple
Red
Yellow
True class
Proportion
1.00
0.75
0.50
0.25
0.00
C
Flesh colour (RF, 5-fold CV)
Predicted class
Yellow
Red
Purple
0.00
0.00
1.00
0.00
1.00
0.00
1.00
0.00
0.00
Purple
Red
Yellow
True class
Proportion
1.00
0.75
0.50
0.25
0.00
