# Supplementary material for: Tissue-Resolved Metabolomic Profiling of Ten Prunus salicina × P. armeniaca Cultivars Reveals Peel–Flesh Metabolic Differences Associated with Fruit Color and Antioxidant Capacity
Source: Foods. 2026 Aug 9;15(16):2790. doi: 10.3390/foods15162790 (PMC13512046; doi:10.3390/foods15162790)
Supplement: Supplementary file 1 [file foods-15-02790-s001.zip › Figure S6.pdf]

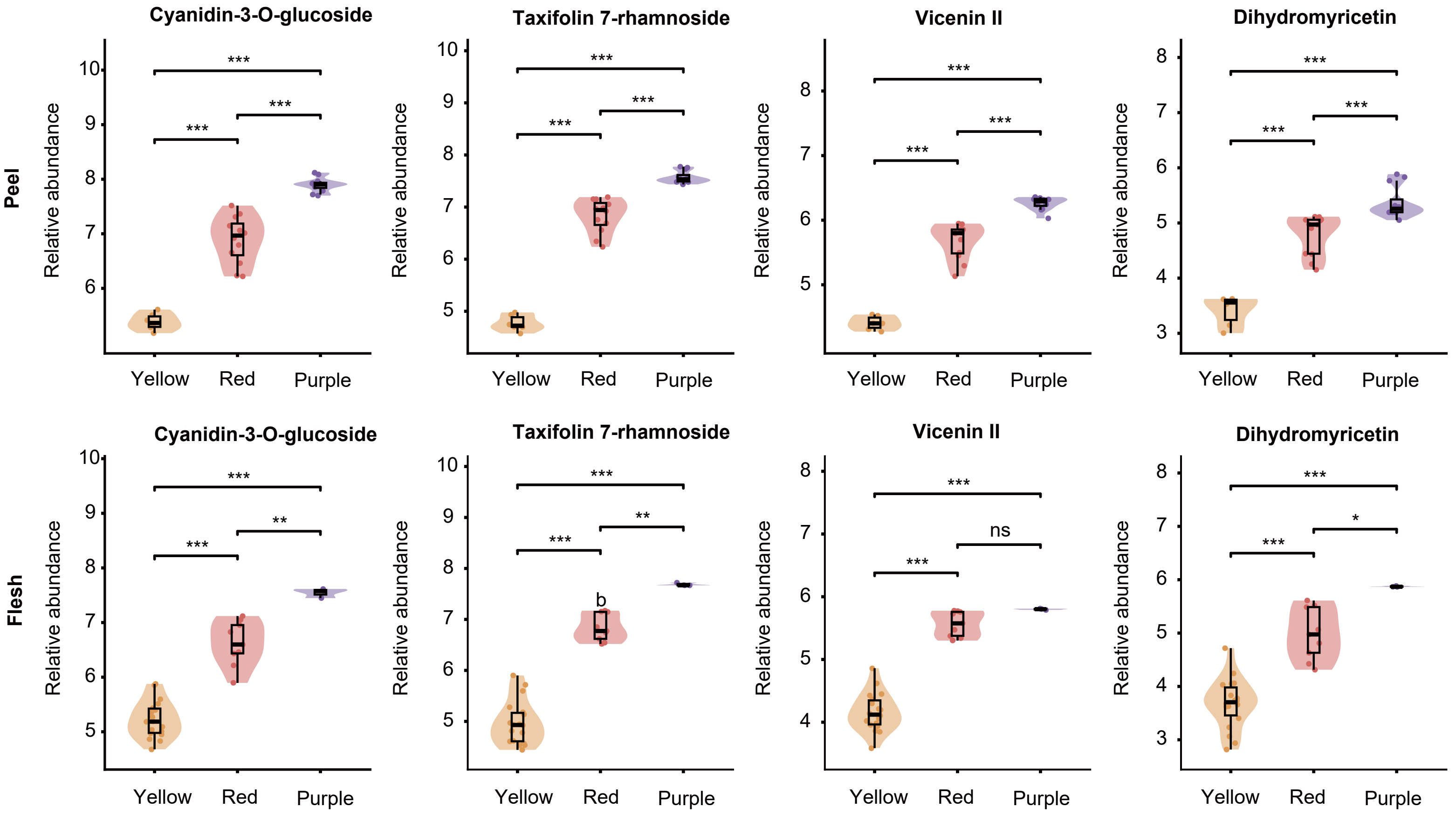
Peel
Flesh
Cyanidin-3-O-glucoside
Taxifolin 7-rhamnoside
Vicenin II
Dihydromyricetin
Relative abundance
Yellow
Red
Purple
***
**
*
ns
b
